# Genomic Standards Consortium Projects

**DOI:** 10.4056/sigs.5559680

**Published:** 2014-02-15

**Authors:** Dawn Field, Peter Sterk, Renzo Kottmann, J. Wim De Smet, Linda Amaral-Zettler, Guy Cochrane, James R. Cole, Neil Davies, Peter Dawyndt, George M. Garrity, Jack A. Gilbert, Frank Oliver Glöckner, Lynette Hirschman, Hans-Peter Klenk, Rob Knight, Nikos Kyrpides, Folker Meyer, Ilene Karsch-Mizrachi, Norman Morrison, Robert Robbins, Inigo San Gil, Susanna Sansone, Lynn Schriml, Tatiana Tatusova, Dave Ussery, Pelin Yilmaz, Owen White, John Wooley, Gregory Caporaso

**Affiliations:** 1 Centre for Ecology and Hydrology, Maclean Building, Benson Lane, Crowmarsh Gifford, Wallingford, Oxfordshire, OX10 8BB, United Kingdom; 2 Oxford e-Research Centre, University of Oxford, Oxford, United Kingdom; 3 Microbial Genomics Group, Max Planck Institute for Marine Microbiology, Bremen & Jacobs University Bremen, Germany; 4 Department of Applied Mathematics and Computer Science, Ghent University, Ghent, Belgium; 5 The Josephine Bay Paul Center for Comparative Molecular Biology and Evolution, Marine Biological Laboratory, Woods Hole, Massachusetts, USA; 6 European Molecular Biology Laboratory (EMBL) Outstation, European Bioinformatics Institute (EBI), Wellcome Trust Genome Campus, Hinxton, Cambridge, United Kingdom; 7 Center for Microbial Ecology, Michigan State University, East Lansing, Michigan, USA; 8 Gump South Pacific Research Station, University of California Berkeley, Moorea, French Polynesia; 9 Biodiversity Institute, Department of Zoology, University of Oxford, The Tinbergen Building, South Parks Road, Oxford, United Kingdom; 10 Department of Microbiology and Molecular Genetics, Michigan State University, East Lansing, Michigan, USA; 11 Institute for Genomic and Systems Biology, Argonne National Laboratory, 9700 South Cass Avenue, Argonne, IL, USA; 12 Department of Ecology and Evolution, University of Chicago, 5640 South Ellis Avenue, Chicago, IL, USA; 13 Information Technology Center, The MITRE Corporation, Bedford, Massachusetts, USA; 14 DSMZ - German Collection of Microorganisms and Cell Cultures GmbH, Braunschweig, Germany; 15 Howard Hughes Medical Institute, and BioFrontiers Institute, and Department of Chemistry and Biochemistry, University of Colorado, Boulder, Colorado, USA; 16 DOE Joint Genome Institute, Walnut Creek, California, USA; 17 Computation Institute, University of Chicago, Chicago, Illinois, USA; 18 National Center for Biotechnology Information, National Library of Medicine, National Institutes of Health, Bethesda, Maryland, USA; 19 University of Manchester, Oxford Road, Manchester, United Kingdom; 20 University of California San Diego, La Jolla, California USA; 21 LTER Network Office, Department of Biology, University of New Mexico, Albuquerque, New Mexico, USA; 22 Institute for Genome Sciences, University of Maryland School of Medicine, Baltimore, MD, USA; 23 DOE Oak Ridge National Laboratory, Oak Ridge, Tennessee, USA; 24 Center for Microbial Genetics and Genomics, Northern Arizona University, Flagstaff, Arizona, USA.

## Abstract

The Genomic Standards Consortium (GSC) is an open-membership community that was founded in 2005 to work towards the development, implementation and harmonization of standards in the field of genomics. Starting with the defined task of establishing a minimal set of descriptions the GSC has evolved into an active standards-setting body that currently has 18 ongoing projects, with additional projects regularly proposed from within and outside the GSC. Here we describe our recently enacted policy for proposing new activities that are intended to be taken on by the GSC, along with the template for proposing such new activities.

## The Genomic Standards Consortium

The Genomic Standards Consortium (GSC) is an open-membership community working towards the development, implementation and harmonization of standards in the field of genomics. The mission of the GSC is to improve digital descriptions of genomes, metagenomes and gene marker sequences. The GSC started in late 2005 with the defined task of establishing what is now termed the “Minimum Information about any Sequence” (MIxS) standard [1,2]. As an outgrowth of the activities surrounding the creation and implementation of the MixS standard there are now 18 projects within the GSC [3]. These efforts cover an ever widening range of standardization activities. Given the growth of projects and to promote transparency, participation and adoption the GSC has developed a “GSC Project Description Template”. A complete set of GSC Project Descriptions and the template are available on the GSC website. The GSC has an open policy of participation and continues to welcome new efforts. Any projects that facilitate the standard descriptions and exchange of data are potential candidates for inclusion under the GSC umbrella. Areas that expand the scope of the GSC are encouraged. Through these collective activities we hope to help foster the growth of the ‘bioinformatics standards’ community. For more information on the GSC and its range of projects, please see http://gensc.org/.

## Initiating and maintaining a project within the GSC

The GSC Project Description template provides a uniform set of statements covering the purpose of the project, the main contacts, the relationship to other projects in the GSC, and other key details that will help the GSC and the wider community to join and use the work of each project group.

Active projects must update their project descriptions annually and submit them to the GSC Board for review. Over time, the list of GSC projects will evolve as activities are added, merged, split or deprecated as demand dictates. Each project should maintain a balanced set of members, especially when the primary goal is to create consensus-driven data sharing solutions.

Project Chairs and members form the core of the GSC. Chairs of projects/working groups report to the GSC Board and help drive GSC workshops and other events. Project working groups define the strategy of the GSC, plan core GSC workshops and host satellite meetings. All working members of GSC projects are encouraged to use GSC events as their primary working forum.

To initiate a new GSC project, or to bring an existing project into the GSC, one or more “champions” prepare a Project Description and present it to the GSC Board. This starts the process of establishing the project within the GSC. Projects are formally voted into the GSC at annual meetings. All proposed GSC activities are open to challenge and improvement over time, especially because the technologies that underpin data sharing change rapidly. Ideally, a balanced working group will be established for all GSC projects, which should be consensus-driven.

By coming together as a community with a common goal and shared interests, we will strengthen and accelerate work towards the goals of establishing best-practices in the capture and exchange of contextual information. The GSC helps grow an ecosystem of standards, tools and resources for empowering a broad community of researchers to describe and share their data. This in turn accelerates the standardization and exchange of an increasing number of data types, and in the long run, promotes open and facile data sharing, thus resolving a current bottleneck for many projects and investigators.
